# Cancer-associated retinopathy preceding the diagnosis of cancer

**DOI:** 10.1186/s12886-018-0948-2

**Published:** 2018-11-03

**Authors:** Florence Hoogewoud, Pauline Butori, Philippe Blanche, Antoine P. Brézin

**Affiliations:** 1 Department of Ophthalmology, National Referral Center for rare Ocular Diseases, Hôpital Cochin, APHP, Université Paris Descartes, Paris, France; 2 Department of Internal Medicine, National Referral Center for Rare Systemic Autoimmune Diseases, Hôpital Cochin, APHP, Paris, France

**Keywords:** Cancer-associated retinopathy, Uveitis, Paraneoplastic retinopathy, Cancer

## Abstract

**Background:**

The early diagnosis of cancer is of crucial importance and a key prognostic factor. Cancer-associated retinopathy (CAR) can be symptomatic prior to other manifestations directly related to malignant tumors. The aim of this study was to show that, in selected cases, ophthalmic findings are consistent enough with the diagnosis of CAR to trigger investigations aimed at detecting a previously unknown malignancy.

**Methods:**

This was a monocentric retrospective case series performed in a tertiary referral center. Patients with a diagnosis of CAR were included. Diagnosis was based on the clinical presentation, the visual field and electroretinogram alterations. The clinical presentation, visual field testing and electroretinographic results were analyzed as well as the malignancies identified following the diagnosis of CAR. Follow-up data was collected.

**Results:**

Four patients (two men, two women, median age 65.5 years) were included. All patients presented with posterior segment inflammation at initial presentation as well as advanced visual field loss and an extinguished electroretinogram. The best corrected decimal visual acuity was 0.8 or better in both eyes of three patients and decreased to 0.3 OD and O.2 OS in one patient due to a bilateral macular edema. No patient had a previously known history of cancer. Once the diagnosis of CAR was made, investigations aimed at identifying a malignant tumors subsequently led to the diagnosis of two cases of small cell lung tumors, of one prostate carcinoma and of a uterine sarcoma. The treatment of CAR included plasmapheresis, systemic corticosteroids, azathioprine, cyclosporine and periocular or intraocular corticosteroid injections. In all cases the intraocular inflammation resolved, but pigment mottling, diffuse retinal atrophy, optic disc pallor and arterial narrowing were among manifestations observed during the follow-up of the patients.

**Conclusion:**

In selected patients, findings suggestive of CAR can be useful for the early detection of a cancer.

**Electronic supplementary material:**

The online version of this article (10.1186/s12886-018-0948-2) contains supplementary material, which is available to authorized users.

## Background

Cancer-Associated Retinopathy (CAR) is a paraneoplastic, autoimmune retinopathy characterized by diffuse retinal degeneration. CAR is associated with a variety of cancers, among which small cell lung carcinoma are the most frequent [1]. Subacute visual loss and visual field constriction are the usual presenting symptoms. The malignancy associated with a CAR can be diagnosed before or after the onset of the ocular manifestations. Because CAR is rare, the descriptions of the disease are based on small series and case reports. As cancer research progresses with improved treatments and increased survival rates, the long term visual outcome of CAR can now be assessed.

The aim of this study was to highlight that in selected cases, ophthalmic findings are consistent enough with the diagnosis of CAR to trigger investigations aimed at detecting a previously unknown malignancy.

## Methods

A retrospective chart review was performed of CAR patients diagnosed at Cochin University Hospital, a uveitis referral center in Paris, France, from 1994 and 2015. The diagnosis of CAR was based on the observation of an intraocular inflammation, the presence of a tubularvisual field (VF) as well as electroretinography (ERG) testing. All patients had a negative family history of retinitis pigmentosa and underwent an extensive workup to exclude other causes of uveitis. The study was performed in accordance with declaration of Helsinki and approved by the ethics committee of the French Society of Ophthalmology (IRB 00008855 Société Française d’Ophtalmologie IRB#1).

## Results

Four patients met our inclusion criteria: two males and two females, with a median age of 65.5 years (range 58 to 71 years). Presenting complaints were a loss of visual acuity in two cases, VF constriction with photophobia in one case, visual vibrations and floaters in another. The time interval between the first symptoms and the first visit to an ophthalmologist ranges from 2 weeks to 1 month. Subsequently three out of four patients were immediately referred to us and one patient was referred 13 months later.

**Fig. 1:**
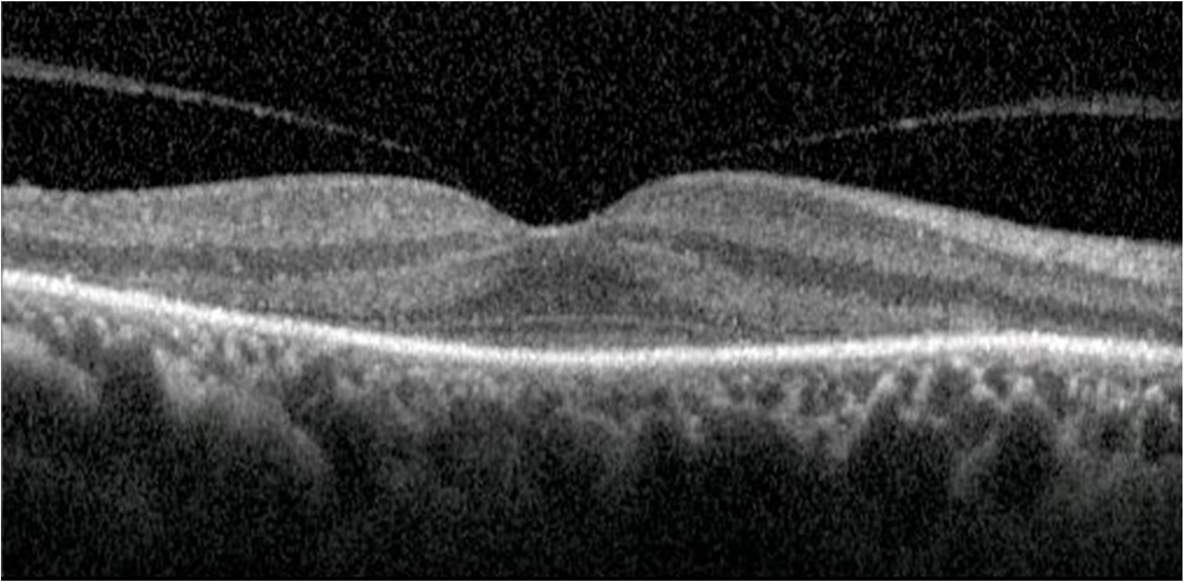
Spectral-domain optical coherence tomography (Spectralis, Heidelberg Engineering, Germany) showing the loss of the external limiting membrane, of the inner segment/outer segment junction and of the outer nuclear and plexiform layers with a sparing of the foveal region

In all patients, the diagnosis of cancer was unknown at the time of the first ophthalmic visit and none had a significant medical history. The initial ophthalmological presentations are outlined in Table 1. Anterior chamber inflammation was absent in three cases and in the other graded 1+ OU without keratic precipitates or synechiae. Posterior segment inflammation included a 1+ vitreous haze and/or peripheral periphlebitis and one case of bilateral macular edema and papillitis. Visual fields were significantly constricted in all cases with a tubular pattern characterized by limits of the V-1 isopter within the central 30°. Two patients had a fluorescein angiography (FA) in the early course of their disease (patients 1 and 3). Both had periphlebitis and papillitis. Patient 3 had additional macular edema. Patient 4 had an FA 7 years after the onset of the disease: she had no active inflammation but a hyperfluorescence around the vessels due to a window defect caused by atrophy. On ERG, three out of four patients had no identifiable A or B waves from the background noise for all stimuli. Patient 3 presented an undistinguishable rod- and mixed-response; cone-response showed severely reduced amplitudes of the A and B waves with normal implicit times and a conserved morphology. Three patients had a best corrected decimal visual acuity (decimal BCVA) of 0.8 or better in both eyes, with a characteristic pattern of outer retinal atrophy and foveolar sparing on OCT (Fig. 1). One patient had a decimal BCVA of 0.3 and 0.2 due to a bilateral cystoid macular edema.

**Table 1:** Patients’ baseline characteristics

Case n°	Gender	Age-range	Type of cancer	Time to diagnosis (months)	Decimal BCVA (OD/OS)	Anterior Segment inflammation	Vitritis	Periphlebitis	Macular edema	Waxy optic pallor	Arteriolar narrowing	OCT	Visual Field	ERG
1	F	61–70	Uterine Sarcoma	2	0.9/1	yes	no	yes	no	yes	yes	ORAFS	tubular	extinguished
2	M	51–60	Lung, small cell	1	0.9/1	no	yes	no	no	yes	yes	ORAFS	tubular	extinguished
3	M	71–80	Prostate adenocarcinoma	25	0.3/0.2	no	yes	no	yes	no	no	CME	tubular	extinguished
4	F	61–70	Lung, small cell	1	0.8/0.8	no	unknown	yes	no	unknown	yes	ORAFS	tubular	extinguished

*BCVA* Best corrected visual acuity, *OCT* Optical coherence tomography, *CME* Cystoid macular edema, *ORAFS* Outer retinal atrophy with foveolar sparing

A cancer was diagnosed in all four patients, with two cases of small cell lung tumor, one case of prostate carcinoma and one uterine sarcoma. The treatment prescribed to the patients included plasmapheresis and/or systemic corticosteroids and/or azathioprine and/or cyclosporine (Additional file 1). Patient 3, who presented with a macular edema and grade 1+ vitritis, was initially diagnosed as a case of idiopathic posterior uveitis and treated by periocular or intraocular corticosteroid injections.

**Fig. 2:**
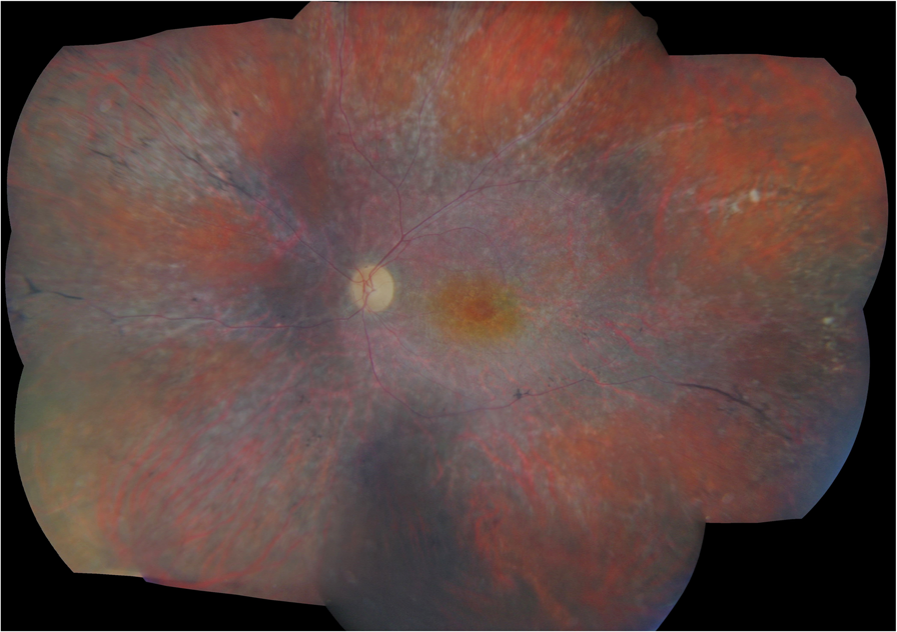
Fundus photography (Digital Non-Mydriatic Retinal Camera, Canon, USA) of patient four at the last follow-up. Diffuse retinal atrophy combined with arteriolar attenuation, optic disc pallor and pigment deposits are observed

Intraocular inflammation subsided in all patients within their follow-up, which ranged from 2 months to 10 years. Patients 3 and 4, for whom the follow-up was the longest, developed a retinitis pigmentosa-like pattern of retinal pigment spicules, diffuse peripheral retinal atrophy and arterial narrowing (Fig. 2). Within the follow-up OCT imaging showed an outer retinal atrophy with foveal sparing in 3 cases, whereas one patient had a diffuse macular atrophy in the left eye and an irreversible macular edema in the right eye.

## Discussion

CAR was first defined in 1976 by Sayer et al. who described a clinical triad of photosensitivity, attenuated retinal arterioles, and visual field loss with a ring scotoma in three patients with non-ophthalmic anaplastic tumor [2]. Subsequently, various case reports have associated this syndrome with other malignancies [3, 4]. Additional clinical features have been described including extinguished ERG, waxy optic disc pallor [5, 6], and retinal vasculitis [7]. Intraocular inflammation as a manifestation of CAR has been previously reported in the literature [8] and was observed in all of our cases at presentation. The intraocular inflammation can lead to an initial work-up targeted to detect other causes of uveitis, before CAR is diagnosed. Optic disc pallor was already seen at the first examination in two patients for whom the time interval between reported symptoms and diagnosis was short. This might reflect the fact that an underlining subacute inflammation might have remained unnoticed for a long time before the first symptoms. Atrophy of the outer retinal layers with foveal sparing was the most frequent OCT finding in our patients and was a helpful sign in the diagnosis of CAR [9]. After an initial inflammatory phase, late findings including retinal pigment epithelial mottling and retinal atrophy have been reported and were observed in our two patients with a long follow-up [10, 11]. In these cases the treatments used were unsuccessful to prevent a diffuse retinal atrophy with a poor visual outcome.

The pathophysiology of CAR remains incompletely understood but molecular mimicry is the generally accepted mechanism. Multiple anti-retinal antibodies have been described in CAR. The most commonly identified auto-antibody is targeted against recoverin. Photoreceptors apoptosis induced by the intravitreal injection of anti-recoverin has been shown in an experimental model [12]. Recent evidence suggests that the cellular rather the humoral immunity can play a role in the disease [13]. This would explain the large number of CAR for which auto-antibodies are not detected. The role of the auto-antibodies as an adjunctive test in the diagnosis of CAR is also controversial with an estimated sensitivity of 55.6% at presentation [5]. The specificity is also low as anti-retinal antibodies can be found in the serum of control patients. [14] Moreover, the absence of auto-antibodies has a very low negative predictive value and the lack of a standardized methods has led to an important variability in the ability of laboratories to detect retinal auto-antibodies [15]. Although retinal auto-antibodies were not tested in our patients, their diagnosis of CAR were based on distinctive ophthalmological manifestations and led us with sufficient confidence to search for a malignancy.

## Conclusion

CAR can precede the diagnosis of cancer. When CAR is recognized ophthalmologists can help their patients in referring them for a workup aimed at detecting a primary tumor.

## Additional files

Additional file 1: Treatment modalities and follow-up data. Description of data: This table describes the treatments and the clinical description at last follow-up. (PDF 43 kb)

## Abbreviations

BCVA: Best-corrected visual acuity
CAR: Cancer-associated retinopathy
ERG: Electroretinogram
OCT: Optical coherence tomography
VF: Visual field
