# Supplementary material for: Cancer-associated retinopathy preceding the diagnosis of cancer
Source: BMC Ophthalmol. 2018 Nov 3;18:285. doi: 10.1186/s12886-018-0948-2 (PMC6215635; doi:10.1186/s12886-018-0948-2)
Supplement: Supplementary file 1 — Treatment modalities and follow-up data. Description of data: This table describes the treatments and the clinical description at last follow-up. (PDF 43 kb) [file 12886_2018_948_MOESM1_ESM.pdf]

Supplementary Table: Treatment modalities and follow-up data

Case n°	Treatment for ophthalmological condition		Follow-up (months)	Decimal BCVA (OD/OS)	Retinal pigment	Peripheral retinal atrophy	OCT (OD/OS)	Visual Field
	systemic	local						
1	Plasmapheresis	-	6	0.7/0.7	no	no	ORAFS/ ORAFS	Ring scotoma stable
2	Intravenous methylprednisolone, Oral prednisone	-	2	1.2/1.2	no	no	ORAFS/ ORAFS	Tubular stable
3	Oral prednisone, azathioprine	3 IVT and 1 sub-Tenon triamcinolone injection	36	CF/0.2	yes	yes	CME/DMA	Ring scotoma worse
4	Plasmapheresis, Intravenous immunoglobulins, azathioprine, oral prednisone, cyclosporine	-	120	0.4/0.2	yes	yes	ORAFS/ ORAFS	Tubular wore

BCVA: Best corrected visual acuity; CF: counting fingers; IVT: intravitreal injection; OCT: Optical coherence tomography; CME: Cystoid macular edema; DMA: Diffuse macular atrophy; ORAFS: Outer retinal atrophy with foveolar sparing
